# Integrated photodetectors for compact Fourier-transform waveguide spectrometers

**DOI:** 10.1038/s41566-022-01088-7

**Published:** 2022-10-24

**Authors:** Matthias J. Grotevent, Sergii Yakunin, Dominik Bachmann, Carolina Romero, Javier R. Vázquez de Aldana, Matteo Madi, Michel Calame, Maksym V. Kovalenko, Ivan Shorubalko

**Affiliations:** 1 Department of Chemistry and Applied Biosciences, ETH Zurich, Zurich, Switzerland; 2 Transport at Nanoscale Interfaces Laboratory, Swiss Federal Laboratories for Materials Science and Technology (Empa), Duebendorf, Switzerland; 3 Laboratory for Thin Films and Photovoltaics, Swiss Federal Laboratories for Materials Science and Technology (Empa), Duebendorf, Switzerland; 4 Grupo de Investigación en Aplicaciones del Láser y Fotónica, Universidad de Salamanca, Salamanca, Spain; 5 Optics and Photonics Technology Laboratory, Ecole Polytechnique Fédérale de Lausanne (EPFL), Neuchâtel, Switzerland; 6 European Space Agency (ESA), European Space Research and Technology Centre (ESTEC), Noordwijk, The Netherlands; 7 Department of Physics and Swiss Nanoscience Institute, University of Basel, Basel, Switzerland; 8 Present Address: Department of Chemistry, Massachusetts Institute of Technology, Cambridge, MA USA

**Keywords:** Optical spectroscopy, Quantum dots, Nanoscale devices, Quantum dots, Optoelectronic devices and components

## Abstract

Extreme miniaturization of infrared spectrometers is critical for their integration into next-generation consumer electronics, wearables and ultrasmall satellites. In the infrared, there is a necessary compromise between high spectral bandwidth and high spectral resolution when miniaturizing dispersive elements, narrow band-pass filters and reconstructive spectrometers. Fourier-transform spectrometers are known for their large bandwidth and high spectral resolution in the infrared; however, they have not been fully miniaturized. Waveguide-based Fourier-transform spectrometers offer a low device footprint, but rely on an external imaging sensor such as bulky and expensive InGaAs cameras. Here we demonstrate a proof-of-concept miniaturized Fourier-transform waveguide spectrometer that incorporates a subwavelength and complementary-metal–oxide–semiconductor-compatible colloidal quantum dot photodetector as a light sensor. The resulting spectrometer exhibits a large spectral bandwidth and moderate spectral resolution of 50 cm^−1^ at a total active spectrometer volume below 100 μm × 100 μm × 100 μm. This ultracompact spectrometer design allows the integration of optical/analytical measurement instruments into consumer electronics and space devices.

## Abstract (web summary)

A Fourier-transform waveguide spectrometer is demonstrated by using HgTe-quantum-dot-based photoconductors with a spectral response up to a wavelength of 2 μm. The spectral resolution is 50 cm^–1^. The total active spectrometer volume is below 100 μm × 100 μm × 100 μm.

## Main

Miniaturization of infrared spectrometers will lead to their wider use in consumer electronics—such as mobile phones enabling food control, the detection of hazardous chemicals and wearable electronics. Besides the highly interesting infrared fingerprint and functional group regions (2.5–20.0 μm), a Fourier-transform near-infrared spectrometer operating between 0.76 and 2.50 μm can be used for the counterfeit detection of medical drugs^1^, or the detection of Earth’s greenhouse gases such as methane and CO_2_ (ref. ^2^); however, higher sensitivity and specificity are usually achieved in the mid-wave infrared region. Furthermore, ultracompact spectrometers are also highly desired for space applications such as femtosatellites (space devices with a maximum weight below 100 g)^3^ and can be useful for novel snapshot hyperspectral cameras (each pixel of a camera consists of an individual spectrometer) requiring ultracompact spectrometers for each pixel to achieve a small pixel pitch^4,5^.

Extensive miniaturization efforts on various elements of spectrometers such as dispersive elements, narrow band-pass filters and Fourier-transform and reconstructive spectrometers have been demonstrated, thoroughly compared in another review article^3^. However, the scaling of spectrometers, so far, comes at a tradeoff between spectral bandwidth, resolution and/or limitation to the visible spectral range^6–8^. Fourier-transform infrared spectrometers combine large spectral bandwidth and resolution in the infrared, but they have not been fully miniaturized yet. Although the interferometric platform of Fourier-transform spectrometers has been downscaled, for example, as optical waveguide spectrometers with impressively high spectral resolution, bandwidth and operation in the infrared region, they still rely on an external imaging sensor for signal detection^9,10^. This means that currently, the overall waveguide spectrometer cannot be smaller than commercially available detectors, which are bulky and expensive for highly sensitive infrared cameras. Although the idea of addressing the challenge of further waveguide spectrometer miniaturization by the monolithic integration of subwavelength photodetectors on top of a waveguide spectrometer is not new^3,9^, it has yet to be realized.

Subwavelength, infrared photodetectors rely on non-scalable device fabrication^11^ or require cryogenic cooling (expensive and bulky)^12^. The scaling of commercial infrared detectors such as InGaAs and mercury cadmium tellurides down to subwavelength dimensions and their integration with optical waveguides is challenging. However, notably, infrared photodetectors based on solution-processable colloidal quantum dots (QDs) offer distinct opportunities: they can be fabricated on various substrates, and the spectral response can be tuned by the QD size and composition. For example, the absorption spectrum of mercury telluride (HgTe) QDs can cover the visible and infrared light region approaching the terahertz region by varying the QD size^13–15^. HgTe-QD-based photodetectors are typically fabricated either as photoconductors or photodiodes and, to the best of our knowledge, have not been monolithically integrated into waveguide spectrometers.

Here we demonstrate the fabrication of a waveguide-integrated, HgTe-QD-based photoconductor. The room-temperature-operated photodetector exhibits a spectral response up to a wavelength of 2 μm. Furthermore, the wire-shaped, subwavelength-sized photodetector was monolithically integrated with an optical waveguide realizing a proof-of-concept Fourier-transform micro-spectrometer with a spectral resolution of 50 cm^−1^ at an active spectrometer volume below 100 μm × 100 μm × 100 μm. This work demonstrates an ultracompact short-wave spectrometer design combining large bandwidth and moderate spectral resolution with a spectral sensitivity in the infrared light region.

**Fig. 1:**
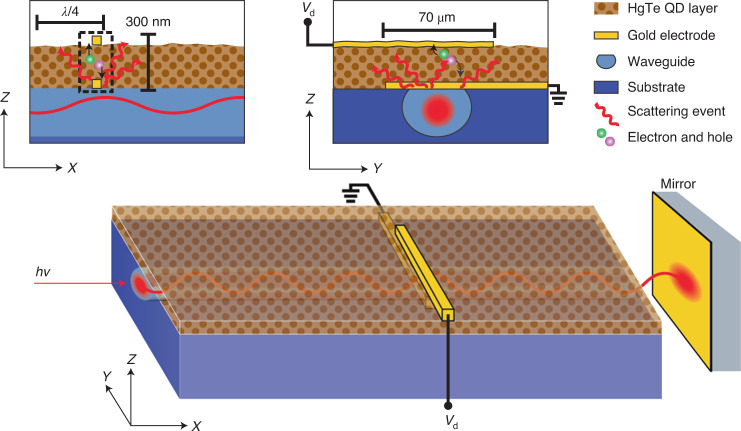
Schematic of a waveguide spectrometer (not to scale). Waveguide spectrometer with a monolithically integrated photoconductor and the respective cross sections. The subwavelength photodetector, fabricated on top of a buried and leaky optical waveguide, consists of one bottom gold electrode functioning as a scattering centre, a photoactive HgTe colloidal QD layer and a top gold electrode. Incoming light is indicated with symbols *h*ν, energy of photons.

**Extended Data Fig. 1:**
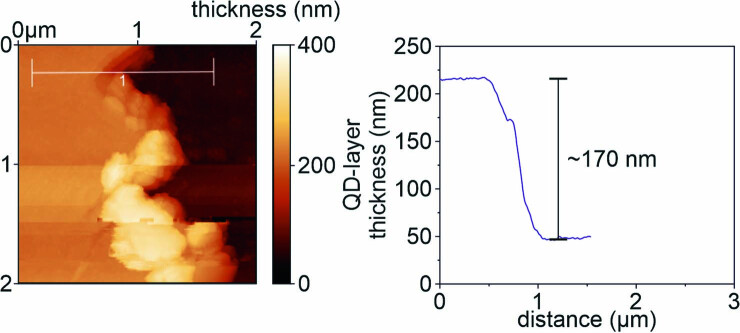
Atomic force microscopy image. The HgTe QD film was partially removed close to the device and the thickness of the film was measured. The profile shows a thickness of 170 nm.

**Extended Data Fig. 2:**
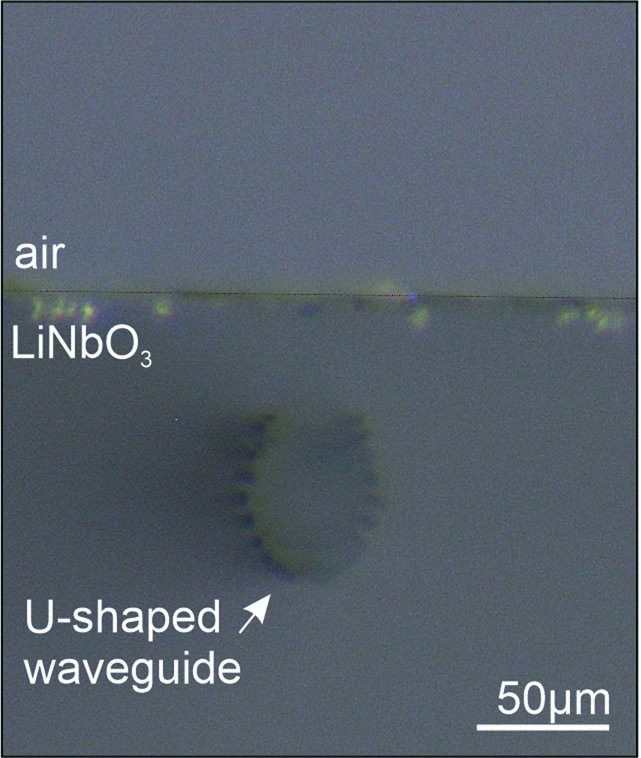
Front-view bright-field optical image of the waveguide. A buried waveguide was laser-written into a LiNbO_3_ substrate.

The schematic in Fig. 1 shows the miniaturization of the detection scheme by monolithically integrating the optical sensor on top of a waveguide. The multimode waveguide was inscribed in a LiNbO_3_ substrate with a depressed-index cladding structure fabricated by femtosecond laser irradiation. In the experiments, only the fundamental mode has been excited by carefully aligning the laser input with the waveguide. The flat substrate surface on top of the buried waveguide enables the deposition of electrodes without risking discontinuity of the electrodes compared with silicon-based waveguides, often having an elevated waveguide on top of the substrate. The specifically designed shape of the waveguide cladding confines near-infrared light but intentionally increases the power leakage along the *Z* direction to increase interactions with the detectors on the surface. Within the waveguide, a stationary wave is created by back-reflection of the waveguide-coupled light at a mirror surface (or by the superposition of two counterpropagating waves entering at both ends of the waveguide). In typical waveguide spectrometers, metal nanorods in proximity to the waveguide probe the intensity profile of the stationary wave and scatter the light proportionally to the local intensity of the stationary wave. The scattered light is subsequently imaged by a commercial camera^9,10,16–20^. Here a detector consisting of a gold bottom electrode with a subwavelength dimension perpendicular to the waveguide and of a length reaching over the complete width of the waveguide is fabricated instead of metal nanorods. For a proof-of-concept device, a HgTe QD photoconductor type was chosen as no band alignments of QD layers are required. The photoconductor was fabricated in a vertical-stacked configuration (as typically used for photodiodes), reducing the footprint area of the sensor. In analogy to metal nanoprobes utilized in typical waveguide spectrometers^9^, the bottom electrode in the photodetector scatters out light from the evanescent field of the stationary wave (that is, simultaneously functioning as an electrode and light scatterer). Subsequently, the light is partially absorbed in the HgTe QD layer creating photoinduced electron–hole pairs. These charge carriers are separated by an applied electric field, resulting in a photocurrent. As the detector is placed just in the evanescent field of the waveguide, the detector can only absorb a fraction of light intensity. This is very well desired as it allows the creation of a stationary wave within the waveguide, which requires that the reflected light intensity is comparable to the incident light intensity. QDs within the evanescent field of the waveguide but outside the photoconductor structure probably lead to some parasitic light absorption reducing the overall light intensity. The parasitic light absorption probably does not contribute to the photoconductor device signal, as the high resistivity of the QD film limits the charge diffusion from neighbouring HgTe QDs. The total QD photodetector thickness, including both electrodes, is below 300 nm (atomic force microscopy measurement of QD layer thickness is shown in Extended Data Fig. 1). Downscaling of the vertical dimension of the imaging sensor by a factor of 1,000 is achieved compared with state-of-the-art waveguide spectrometers using external InGaAs cameras and appropriate optics (typically resulting in a thickness of around 30 cm). Adding the thickness of the buried, leaky waveguide (front view of the optical bright-field image is shown in Extended Data Fig. 2) to the QD photoconductor thickness results in an overall spectrometer device thickness below 100 μm. With an overlap of the electrodes of 70 μm and a mirror travel range of 100 μm, the resulting dimensions of the ultracompact spectrometer are below 100 μm × 100 μm × 100 μm, which includes the optical system and imaging sensor (but excludes the electrical circuit). We have excluded the piezoelectric stage from the spectrometer volume calculation, as other device architectures without a moving mirror exist, for example, a stationary wave created by counterpropagating waves^10^ or with a fixed mirror position and an array of nanoprobes^20^. In this proof-of-concept research study, a single photodetector was characterized, which requires a phase modulation, for example, utilizing a piezo-stage-mounted movable mirror.

**Fig. 2:**
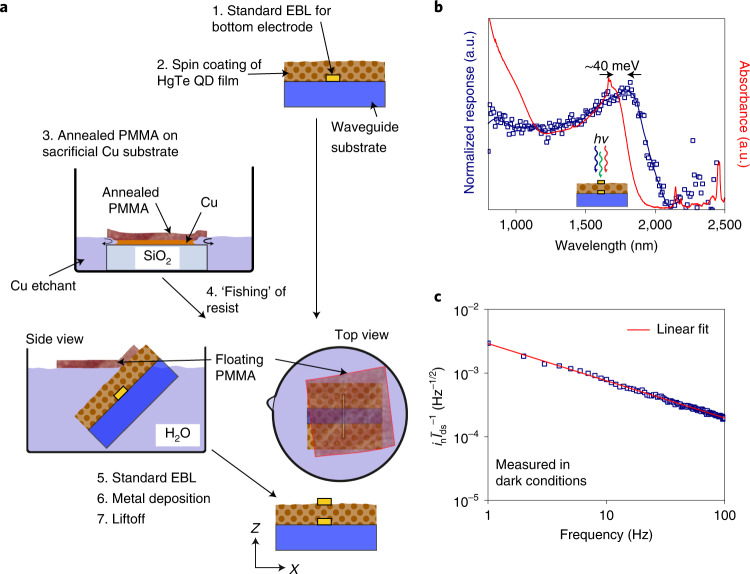
Basic photodetector fabrication and characterization. **a**, Schematic of the detector fabrication showing a wet chemical transfer of annealed PMMA on top of the QD thin film. **b**, Comparison of the photoresponse (illuminated from top, blue squares) with the HgTe QD absorption spectrum in solution (red line). **c**, Noise current of the respective photodetector. Source data

**Extended Data Fig. 3:**
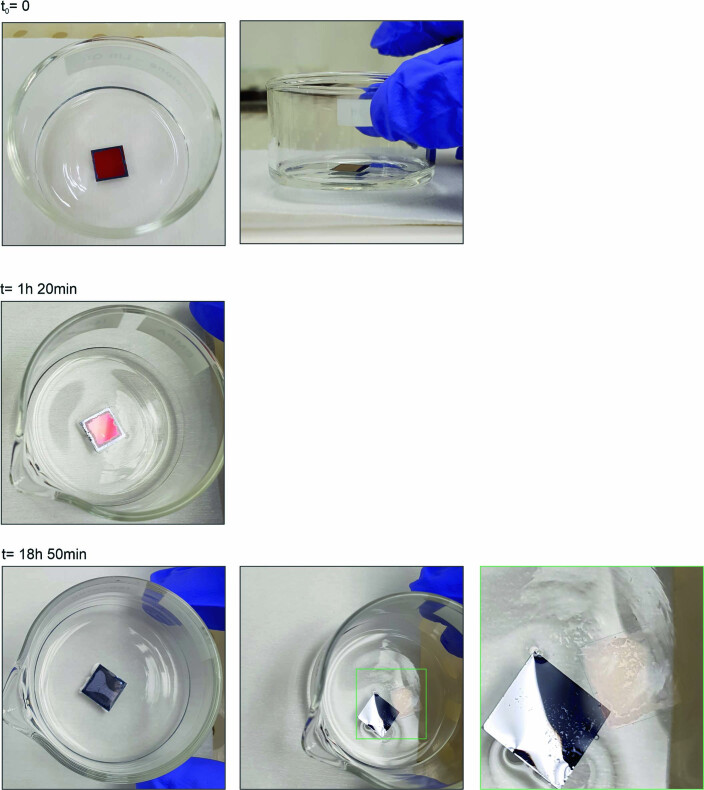
Optical images of the PMMA transfer preparation. The edges of the PMMA and Cu layer were mechanically removed to give access for the Cu etchant solution to the Cu layer. After 1 h 20 min, the Cu layer is already clearly etched at the edges. After 18 h 50 min, the Cu is fully etched and the PMMA is floating on the Cu etching solution.

Figure 2a illustrates the fabrication process of the detector (detailed description is provided in the Methods section). The bottom electrode is fabricated with standard electron-beam lithography. The HgTe QD film is deposited in a layer-by-layer manner by alternating spin-coating steps of QD dispersion followed by a ligand exchange solution containing 1,2-ethanedithiol. The fabrication of a suitable top electrode with submicrometre dimensions on top of a QD film is challenging and, to the best of our knowledge, not developed yet. For example, lithography via a shadow mask lacks alignment precision and results in feature sizes considerably larger than 1 µm. Furthermore, standard high-resolution electron-beam lithography requires the deposition of a resist such as poly(methyl methacrylate) (PMMA) by spin coating and subsequent annealing of the resist typically at 180 °C. Unfortunately, the heating of most QD films to such high temperatures compromises the structural and chemical integrity of QDs (for example, oxidation). A workaround, inspired by graphene transfer, prepares the PMMA double layer by spin coating and annealing it on a 100 nm copper buffer layer on a SiO_2_ substrate. This stack was placed in a beaker containing a copper etchant solution (optical images are shown in Extended Data Fig. 3). Overnight and starting from the edges, the copper is completely etched due to the capillary forces of the solution. Once the copper is completely etched, the PMMA detaches and floats at the liquid/air interface. After rinsing of the already annealed PMMA sheet, it is picked up with the half-fabricated HgTe QD photoconductor by fishing it from the liquid/air interface. After drying at ambient conditions, the sample can be treated with standard electron-beam lithography. Further development may use roll-to-roll PMMA transfer by lamination from a donor substrate with a low-adhesive surface treatment. Importantly, the possibility of detector fabrication is not limited to LiNbO_3_ substrates, but can be extended to various flat substrates including SiO_2_ on silicon, demonstrating the compatibility with complementary metal–oxide–semiconductor on-chip integration.

**Extended Data Fig. 4:**
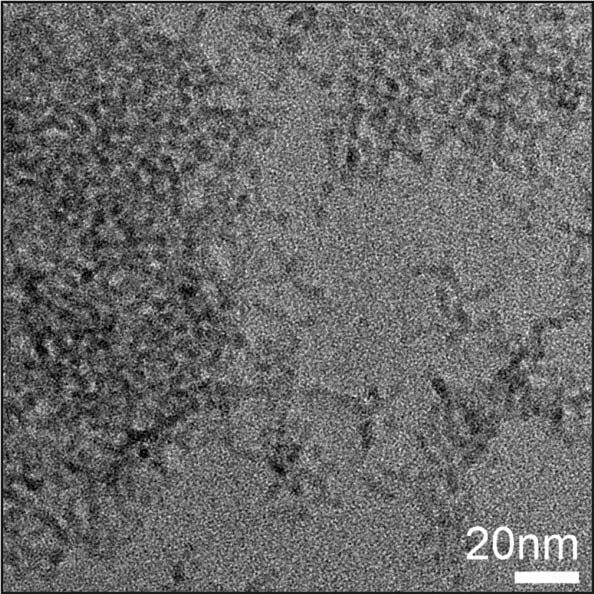
Transmission electron microscopy image of HgTe QDs. The image was recorded with a Tecnai F30 (Thermo Fischer Scientific) instrument with an acceleration potential of 300 kV (field emission gun).

Figure 2b shows the absorbance of tetrapodic-shaped HgTe QDs (transmission electron microscopy image is shown in Extended Data Fig. 4) in solution and compares it with the photoresponse of a corresponding HgTe QD photoconductor consisting of a compact QD film. The spectral dependence of the photoresponse is acquired by illuminating the device from top in a monochromator setup. A bathochromic shift of about 40 meV is observed, which is expected due to the increased electronic coupling of the QDs in films and after ligand exchange with shorter ligands. The detector exhibits a spectral photoresponse in the infrared comparable to commercially available InGaAs photodetectors with a quantum-confined excitonic peak at 1.8 μm. Figure 2c shows the noise current of the detector measured in the dark, exhibiting a 1/*f* noise behaviour commonly observed in photoconductors^21–24^. The active device area depends on the transparency of the thin gold electrodes and the extension of the electric field (applied between the electrodes) reaching into the QD film. Although the gold electrodes overlap over a length of 70 μm and a width of 60 nm, contributions to the photocurrent may mainly originate from the edges of the device and only limited contributions may come from photons reaching the HgTe QD film through the 60-nm-thick gold electrode. For an estimation of the photoresponsivity and specific detectivity, the overlap of the electrodes can be used as the device area resulting in 0.6 A W^–1^ and 3 × 10^9^ Jones, respectively (a detailed calculation is provided in the Methods section). However, the active device area may substantially differ when the detector is illuminated (from the side) through the waveguide, and the photosignal may benefit from plasmonic field enhancement of the subwavelength device structure.

**Fig. 3:**
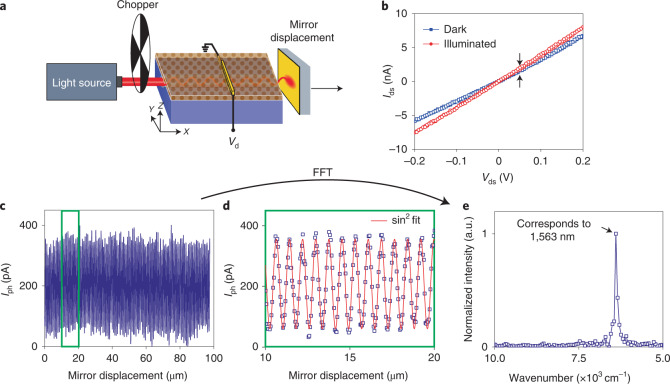
Spectrometer application with a 1,570 nm laser light source. **a**, Schematic of the spectrometer experiment. The photosignal is modulated by a mechanical chopper (27 Hz) and the interferogram is measured as a function of mirror displacement (step size, 50 nm) (not to scale). **b**, Current (*I*_ds_)–voltage (*V*_ds_) characteristics of the photoconductor in dark and under illumination through the waveguide. **c**, Photosignal as a function of mirror displacement. **d**, Zoomed-in view of the acquired photosignal in **c**. **e**, Corresponding fast Fourier transformation (FFT) of the photosignal in **c**. Source data

**Extended Data Fig. 5:**
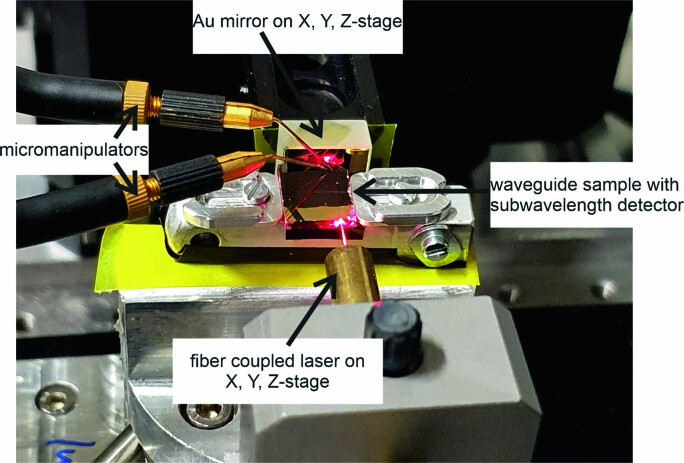
Optical image of the setup. A red alignment laser was used to visualize the beam path from the fibre into the LiNbO_3_ waveguide/sample and reflection at a gold mirror. Two micromanipulators were used to contact the subwavelength photoconductor. They were brought into place by viewing their approach to the contact pads by an optical camera recording from the top (the camera is not visible in this picture).

**Extended Data Fig. 6:**
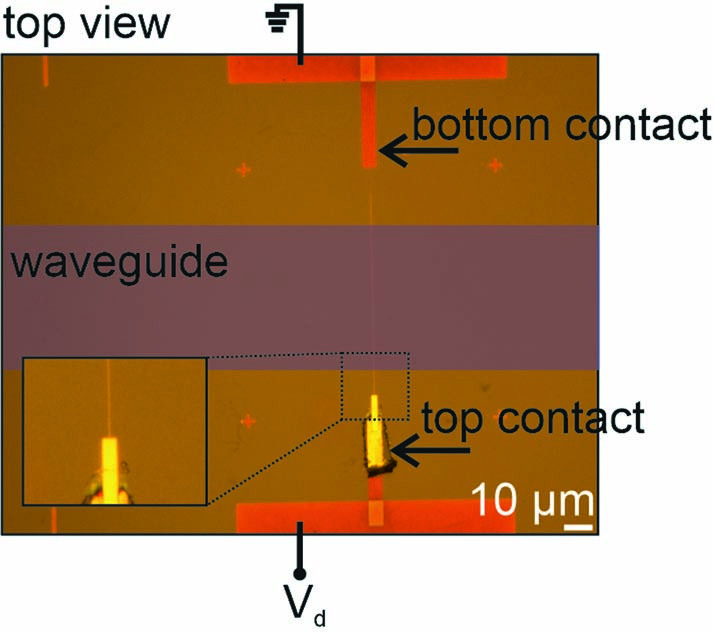
Top-view bright-field optical image of the subwavelength photoconductor. Two gold contact pads are partially depicted at the top and bottom of the image. The colour of the gold appears reddish as the sample is coated with a 170 nm thick HgTe QD film. On the lower contact pad, the QD film is removed in an area to connect the contact pad to the top electrode. The top electrode appears in a golden colour. The 60 nm wide electrode is visible at high magnification in the inset due to light scattering at subwavelength objects.

**Extended Data Fig. 7:**
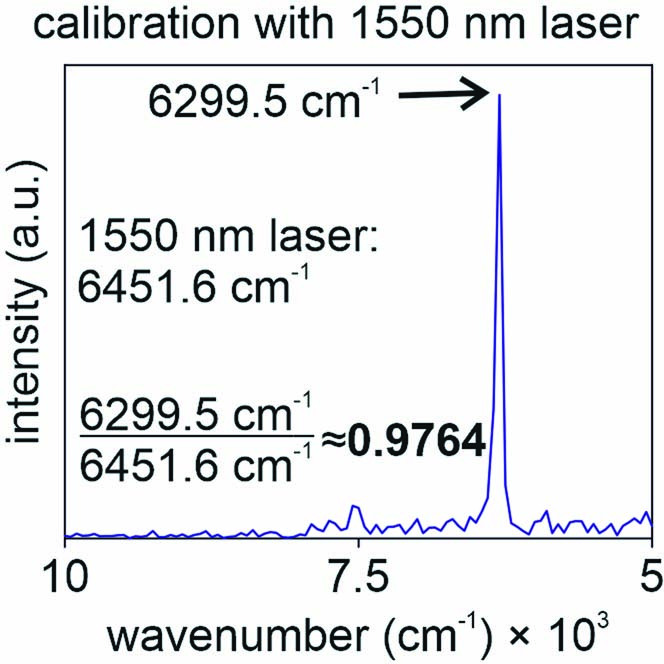
Calibration of the piezo stage. The piezo stage was calibrated with the spectrometer presented in this paper. A laser with a wavelength of 1550 nm was coupled into the spectrometer and the photo signal of the stationary wave was recorded over 100 μm mirror displacement (piezo travel range). A fast Fourier transformation resulted in a slight offset from the expected value giving rise to a calibration value of 0.9764. Therefore, the real piezo stage travel distance was 97.64 μm. Source data

A schematic of the setup is shown in Fig. 3a (optical image of the setup is shown in Extended Data Fig. 5 and an optical image of the device is shown in Extended Data Fig. 6): a 1,570 nm laser (300 μW output power) is modulated by a mechanical chopper (50% open areas) and coupled into a LiNbO_3_ waveguide. The modulation of light by the chopper allows to recover the signal with a low-noise lock-in amplifier. A stationary wave is created within the waveguide due to the back-propagation of the light reflected from a mirror on the opposite end of the waveguide. Thus, the modulated photosignal depends on the relative position of the subwavelength photodetector: it gives a maximum of the photosignal at antinode positions and a minimum photosignal at node positions of the stationary wave. A slight decrease in conductivity was observed within the first week (after device fabrication), which stabilized afterwards, giving a constant conductivity over the course of at least three months (the sample was stored in an inert atmosphere in between measurements). The current–voltage curves (Fig. 3b) show an ohmic device behaviour with a resistance of about 30 MΩ once a stabilized device performance was observed with a notably increased conductivity under illumination. The mirror is mounted on a piezostage and the travel range along the waveguide axis is calibrated by using the waveguide spectrometer as an interferometer with a calibration wavelength of 1,550 nm (Extended Data Fig. 7). For spectroscopic experiments, the calibrated mirror is moved away from the waveguide increasing the optical path difference. The stationary wave shifts with respect to the mirror position and the subwavelength photodetector goes through nodes and antinodes of the stationary wave. The resulting photosignal is shown in Fig. 3c with a 100 μm mirror travel range; a zoomed-in view of the results in Fig. 3c is shown in Fig. 3d. A typical mirror scan experiment was acquired in about 160 min and only minor signal fluctuations are observed (Fig. 3c). The corresponding fast Fourier transformation is also shown (Fig. 3e). The wavelength of the coupled laser is well determined with the ultracompact spectrometer. The resolution of the Fourier-transform infrared and waveguide spectrometers is the inverse of the optical path difference (that is, two times the mirror displacement). A travel range of the mirror by 100 μm results in a spectral resolution of 50 cm^−1^ (13 nm resolution at a wavelength of 1,570 nm). In comparison to other spectrometer types, a spectral resolution of 50 cm^−1^ may not be very high; however, all the spectrometers are subject to a tradeoff among the spectral resolution, bandwidth and spectrometer volume. In this regard, the presented waveguide spectrometer reaches the fundamental limits and further miniaturization may require smaller waveguide cross sections. When a higher spectral resolution is desired, a longer optical path difference can be chosen^17,25,26^, which will inherently increase the waveguide length and spectrometer volume. The maximal detectable wavelength of the presented waveguide spectrometer is determined by the absorption spectrum of the HgTe QD film, reaching about 2 μm in our case. The minimal unambiguously detectable wavelength is limited by the transmission of LiNbO_3_ in the ultraviolet region of about 400 nm once the chosen sampling interval satisfies the Nyquist–Shannon sampling theorem.

**Fig. 4:**
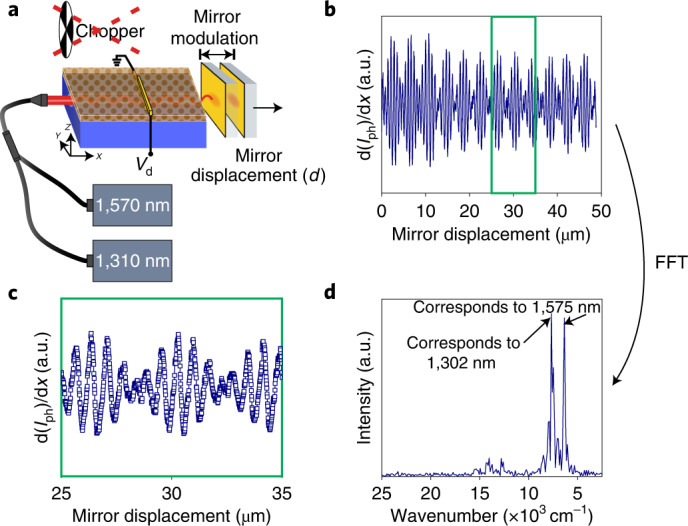
Spectrometer application with the modulation of mirror in addition to mirror displacement. **a**, Schematic of the experiment with two laser light sources coupled simultaneously (1,570 nm, 300 μW; 1,310 nm, 330 μW) (not to scale). **b**, Photosignal as a function of mirror displacement. **c**, Zoomed-in view of the acquired photosignal in **b**. **d**, Corresponding fast Fourier transformation of the photosignal in **b**. Source data

**Extended Data Fig. 8:**
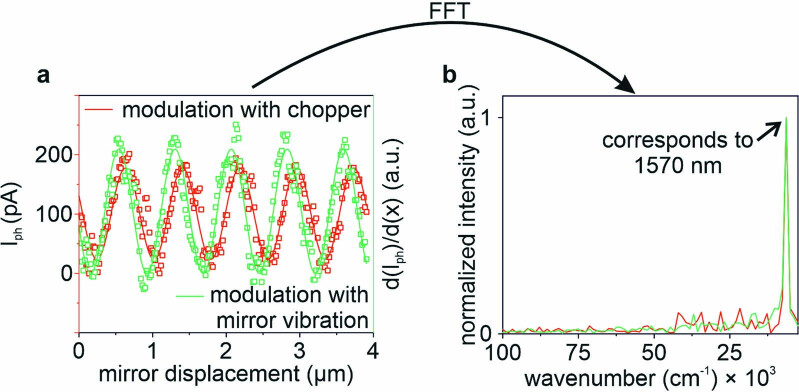
Comparison of the spectrometer with light modulation from a mechanical chopper or modulation from mirror vibration. The modulation with a vibration mirror gives a slightly larger photo signal, however, it strongly depends on the mirror vibration amplitude in respect to the wavelength of the stationary wave. While light modulation with a mechanical 50/50 chopper does remove 50% of the light intensity, it is still present and adds to the photo signal in case of mirror modulation. A fast Fourier transformation of both signals results in the same signal position and comparable noisy background when normalized. Source data

**Extended Data Fig. 9:**
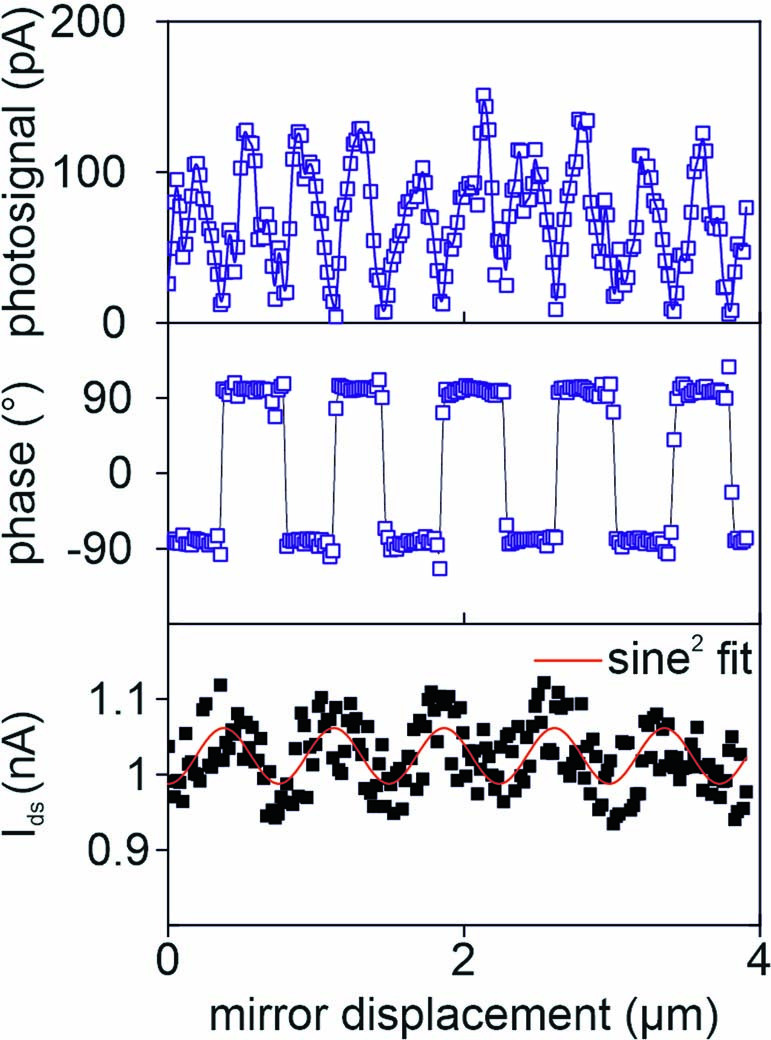
Raw data of the spectrometer with light modulation through the vibration of the mirror. The lock-in can only display positive values, therefore, depending on the curvature of the stationary wave a phase jump of 180° is observed. The photo signal can also be directly obtained from the drain current but at a lower signal strength. Source data

We can simplify the optical waveguide scheme: instead of modulating the waveguide-coupled light with a bulky mechanical chopper or a shutter, the photosignal can also be effectively modulated by periodically vibrating the mirror (independent of mirror displacement). In other words, a.c. modulation is superposed with the d.c.-modulated mirror displacement. In fact, the modulation of the signal by a mechanical chopper or by mirror vibrations leads to comparable results (Extended Data Fig. 8; raw data of the mirror-modulated photosignal are shown in Extended Data Fig. 9). Signal modulation by the vibration of the mirror allows for a more compact spectrometer design and allows us to directly couple multiple laser light sources to the optical waveguide. Figure 4a shows a schematic of the setup with laser light simultaneously coupled from a 1,570 and 1,310 nm laser with optical laser output powers of 300 and 330 μW, respectively. The recorded photosignal is shown in Fig. 4b,c, exhibiting a well-resolved beating pattern and Fig. 4d shows the corresponding fast Fourier transformation of the photosignal. The signal of both lasers is of similar intensity, in agreement with the corresponding laser powers. The spectral resolution is about 100 cm^−1^ (50 μm mirror displacement) translating into 25 and 18 nm resolution at a wavelength of 1,570 and 1,310 nm, respectively.

The monolithic integration of subwavelength infrared photodetectors has a tremendous effect on the scaling of Fourier-transform waveguide spectrometers, but may also be of interest for miniaturized Raman spectrometers, waveguide-based biosensors and lab-on-a-chip devices^27^, as well as the development of high-resolution snapshot hyperspectral cameras^4^.

Further improvements in integrated subwavelength photodetectors may come with the implementation of photodiodes exhibiting higher light sensitivity, and the extension of spectral sensitivity into the mid-wavelength infrared region by utilizing larger HgTe QDs^15,21,22,28,29^ and intraband absorption^30,31^. Furthermore, instead of a single photodetector, a photodetector array, ideally with a subwavelength period, can be fabricated. However, the subwavelength periodic arrays may exhibit electronic and photonic crosstalk between the photodetectors—which can limit the detector periodicity to a period larger than the wavelength. In this case, the integration of a Mach–Zehnder modulator may be useful^10^. Furthermore, the moving mirror can be replaced, for example, with a stationary mirror deposited at the end of a waveguide^20^, or alternatively, by a loop-shaped waveguide design^9^. In addition, PMMA transfer may be further developed towards a roll-to-roll lamination process by adjusting the adhesive strength between the sacrificial substrates and the desired HgTe QD layer.

To summarize, monolithically integrated subwavelength photodetectors are crucial to achieve the full miniaturization potential of Fourier-transform waveguide spectrometers. A fabrication method of such detectors involves introducing circumventing temperature-sensitive processing steps. A subwavelength photodetector based on HgTe QDs has been monolithically integrated onto a LiNbO_3_ waveguide with a spectral sensitivity up to a wavelength of 2 μm and room-temperature operation. The monolithic integration of the photodetector downscales the thickness of the imaging sensor by a factor of 1,000, resulting in a large-bandwidth, ultracompact (below 100 μm × 100 μm × 100 μm) infrared micro-spectrometer with a moderate spectral resolution of 50 cm^−1^. The presented results pave the way towards micro-spectrometers in consumer electronics, space applications and hyperspectral cameras.

## Methods

### Chemicals

Acetone (≥99.5%, Sigma-Aldrich), ammonium persulfate (≥98.0% Sigma-Aldrich), chlorobenzene (99.9%, Sigma-Aldrich), didodecyldimethylammonium bromide (98.0%, Sigma-Aldrich), 1-dodecanethiol (98.0%, ACROS Organics), 1,2-ethanedithiol (≥98.0%, Sigma-Aldrich), hydrochloric acid (37.0%, VWR Chemicals), mercury(ii) chloride (≥99.5%, Sigma-Aldrich), methanol (≥99.9%, Sigma-Aldrich), methyl-iso-butyl ketone (MIBK; 99.8%, VWR Chemicals), oleylamine (>95.0%, Strem Chemicals), tellurium (99.999%, Sigma-Aldrich), anhydrous tetrachloroethylene (≥99.0%, Sigma-Aldrich), tri-*n*-octylphosphine (97.0%, STREM), 50 K PMMA (4.0% in anisole, Allresist), 950 K PMMA (4.5% in anisole, Allresist) and 2-propanol (≥99.8%, Sigma-Aldrich).

All the chemicals were used as received, unless otherwise stated. Oleylamine was dried and degassed under reduced pressure at a temperature of 120 °C for 1 h before its use.

Trioctylphosphine telluride (TOP:Te) was synthesized by stirring 5.08 g tellurium (0.04 mol) in dry trioctylphosphine (20 ml) for three days yielding a 2 M TOP:Te solution. The solution was filtered through a 0.45 μm polytetrafluoroethylene syringe filter resulting in a clear, yellowish solution.

Cleanroom: 21.5 °C; 45–50% relative humidity; particle class, 10–10,000; >18 MΩ cm purified water.

### HgTe QD synthesis

#### Caution Highly toxic compounds

Highly toxic compounds are used and appropriate precautions have to be implemented. The reaction was adapted from another work^13^ with slight modification. Briefly, 27 mg (0.1 mmol) HgCl_2_ and 4 ml oleylamine were heated to 100 °C under inert conditions and under continuous stirring at 1,000 rpm. The reaction was kept for 30 min at 100 °C to ensure the complete solvation of HgCl_2_. Subsequently, the reaction was cooled to 70 °C, and a mixture of 65 μl TOP:Te (0.13 mmol) in 700 μl oleylamine was quickly injected. After 90 s of QD growth, the reaction was quenched in a mixture of 200 μl 1-dodecanethiol, 200 μl tri-*n*-octylphosphine and 4 ml tetrachloroethylene. The HgTe QD dispersion was purified by precipitation of QDs with 6 ml methanol and centrifugation at 4,000 rpm (~2,200 maximum relative centrifugal force) for 1 min. The supernatant was discarded and the QDs were dispersed in 1 ml tetrachloroethylene. Three drops of oleylamine and three drops of 1-dodecanethiol were added to the dispersion followed by the addition of 1 ml acetone and three drops of a mixture of 10 ml 2-propanol and 50 mg didodecyldimethylammonium bromide to increase the QD stability^32^. For QD precipitation, 800 μl methanol was added, followed by centrifugation at 4,000 rpm (~2,200 maximum relative centrifugal force) for 1 min. The supernatant was discarded and the QDs were dispersed in chlorobenzene and filtered through a 0.2 μm polytetrafluoroethylene syringe filter.

### Waveguide fabrication

The waveguide was fabricated by using an amplified Ti:sapphire femtosecond laser system (Spitfire, Spectra Physics): it delivered 120 fs pulses (Fourier-transform limited) at a central wavelength of 795 nm and operated at a repetition rate of 1 kHz. The beam power was finely controlled by a half-wave plate and a linear polarizer, followed by a calibrated neutral density filter and was focused by a ×40 (numerical aperture, 0.65) microscope objective. The sample was placed in a computer-controlled XYZ stage (0.05 μm precision) to scan the sample in the focal region of the objective, describing the desired trajectories to fabricate a ‘U’-shaped cladding consisting of about 30 parallel damage tracks. A mean beam power of 0.17 mW (estimated after the microscope objective) was set as the optimum value for fabrication, and the scanning velocity was set to 500 μm s^–1^ to obtain continuous tracks along the waveguide (good spatial overlap between consecutive pulses) but minimizing the stress induced in the crystal by irradiation.

### Device fabrication

For the bottom electrode fabrication on top of the waveguide, briefly, a PMMA double layer was fabricated by spin coating 50 K PMMA 4% in anisole at 4,000 rpm for 45 s, followed by 3 min annealing at 180 °C. Subsequently, 950 K PMMA 2.25% in anisole was spin coated at 4,000 rpm for 45 s followed by 5 min annealing at 180 °C. Espacer 300Z was spin coated at 4,000 rpm for 45 s and subsequently annealed for 3 min at 110 °C. The desired structures were exposed by electron-beam lithography (30 kV, 10 μm aperture, 4 nm step size, dose of 350 μC cm^–2^). After exposure, the sample was stirred in water for 30 s, developed for 60 s in a mixture of MIBK:2-propanol (1:3), rinsed for 30 s in 2-propanol and dried with a nitrogen drying gun. Subsequently, 2 nm chromium and 40 nm gold were deposited by electron-beam-assisted thermal evaporation. After liftoff at 50 °C in acetone, the sample was rinsed in 2-propanol and dried with a nitrogen drying gun. The sample was cleaned in an oxygen plasma cleaner for 60 s at 1 mbar.

The QD thin film was fabricated by layer-by-layer deposition at ambient conditions: HgTe QD dispersion was spin coated at 3,000 rpm for 45 s, followed by spin coating a ligand exchange solution (10 ml 2-propanol, 200 μl HCl(aq) and 200 μl 1,2-ethanedithiol) at 3,000 rpm for 45 s. Then, 2-propanol was spin coated at 3,000 rpm for 45 s for rinsing the sample. This process was repeated four more times, resulting in a QD layer thickness of 170 nm.

A second substrate was prepared by electron-beam-assisted thermal evaporation of 100 nm copper on top of a Si/SiO_2_ wafer. A PMMA double layer was spin coated on top of copper: 50 K PMMA 4% in anisole at 4,000 rpm for 45 s followed by 3 min annealing at 180 °C. Subsequently, 950 K PMMA 2.25% in anisole was spin coated at 4,000 rpm for 45 s followed by 5 min annealing at 180 °C. After spin coating, the PMMA was scratched away from the edges of the sample. This allows access of the copper etchant solution to the sandwiched copper layer. The PMMA/Cu/SiO_2_/Si sample was placed in a water-based ammonium peroxodisulfate solution (25 g per 100 ml). Overnight, the copper was completely etched away, and the PMMA floated on top of the copper etchant solution. The copper etchant solution was then replaced with ultrapure water. The water was further replaced three more times over 4 h with fresh water to ensure a clean backside of the floating PMMA.

The floating PMMA was fished with the HgTe QD/waveguide sample. The transferred PMMA/HgTe QD/waveguide sample was dried at ambient conditions, which took about 10 min. The design of the top electrode was written by electron-beam lithography (30 kV, 10 μm aperture, 4 nm step size, dose of 350 μC cm^–2^) into the PMMA double layer. After development for 60 s in a developer (MIBK:2-propanol (1:3)), rinsing with 2-propanol and drying with a nitrogen drying gun, 60 nm gold was deposited by electron-beam-assisted thermal evaporation. Subsequently, a liftoff was performed in 50 °C warm acetone; afterwards, the sample was rinsed in 2-propanol and dried with a nitrogen drying gun.

### Waveguide illuminated sample setup

The sample was mechanically fixed on a substrate holder. A free-space laser (1,510–1,587 nm; 6328 tunable diode laser, New Focus) was mechanically modulated with an optical 50/50 chopper and coupled into an optical fibre. The end of the optical fibre was stripped from its cladding, cleaned, cleaved and mounted on an XYZ mechanical stage (M-VP-25 XL, Newport) to align the optical fibre with the LiNbO_3_ waveguide. On the other end of the waveguide, a gold-coated mirror was mounted on a 100-μm-travel-range piezo-stage (P-517.3CL, Physik Instrumente).

The sample was contacted with two micromanipulators and typically biased with 50 mV (2614B, Keithley). The modulated photosignal was amplified over a 100-kΩ-load resistor and measured with a lock-in amplifier (SR860, Stanford Research Systems), receiving the lock frequency from a mechanical chopper (27 Hz). The d.c. output voltage signal of the lock-in amplifier was programmed to deliver a driving voltage for the piezo-stage. One part of the measured photosignal is dependent on the mirror position, whereas the second part is stray light in the sample, which is not dependent on the mirror position. The undesired background was subtracted from the photosignal. The mirror travel distance was multiplied by 2 to obtain the optical beam path difference, followed by fast Fourier transformation.

In the case that two lasers were coupled, a fibre-coupled free-space laser (1,510–1,587 nm; 6328 tunable diode laser, New Focus) and a fibre-coupled laser (1,270–1,330 nm; TLB-6724-P, New Focus) were coupled with a wavelength-division multiplexer (1,310 and 1,550 nm; WD1350A, Thorlabs) into a single optical fibre. Also, in this experiment, the sample was contacted with two micromanipulators and biased with 50 mV (2614B, Keithley). The fibre-coupled light could not be modulated by a mechanical chopper, and therefore, the modulation was performed by vibrating the mirror. The lock-in output voltage was programmed to deliver a driving voltage (d.c. with a sinusoidal a.c. component) for the piezo-stage. The a.c. component of the stage was about 100 nm (r.m.s.). The modulation frequency was also supplied by the lock-in amplifier and we chose 27 Hz. In this experiment, the measured photosignal (measured over 100 kΩ resistor by the lock-in amplifier) is the derivative of the stationary wave due to the a.c. modulation of the stationary-wave position underneath the photodetector. Undesired stray light does not contribute to the overall photosignal as it is not frequency modulated by the mirror or reflected away from the photodetector. As the photosignal oscillates around zero, a phase jump by 180° is observed with the lock-in amplifier. These 180° phase jumps were normalized to jump from 1 to −1 $\left(\delta _{\mathrm{norm}} = \cos\left( {\frac{{(\delta - \delta _0) \times \uppi }}{{180}}} \right)\right)$. The normalized phase was multiplied with the amplitude to give the phase-corrected photosignal. The mirror travel distance was multiplied by 2 to obtain the optical beam path difference, followed by fast Fourier transformation.

### Photoresponse setup

Light from a broad light source was mechanically modulated (MC2000B-EC, Thorlabs) and optically monochromated (SpectraPro HRS-300, Princeton Instruments; gratings, 150 Grooves mm^−1^ and blaze wavelength of 0.8 μm; 150 Grooves mm^−1^ and blaze wavelength of 2.0 μm). Higher-frequency orders were filtered out with long-pass filters (780, 1,000 and 1,500 nm). The beam was collimated and divided into a reference beam characterized by a reference detector (UM-9B-L, Gentec-EO) and an illumination beam for the sample. The sample was biased with 50 mV (2614B, Keithley) and the photosignal was measured over 100-kΩ-load resistance with a lock-in amplifier (SR865, Stanford Research Systems).

### Noise setup

The sample was biased with a battery-powered current amplifier (SR570, Stanford Research Systems), and the drain current was amplified with the same amplifier and subsequently measured with a data acquisition board (USB-6281, National Instruments) at a sampling rate of 625 kHz. A 40 kHz low-pass filter was employed. The d.c.-current offset of the drain current was removed, and power spectral densities of 385 one-second-long time traces were calculated and subsequently averaged. The 50 Hz net frequency was manually removed from the result.

### Calculation of responsivity and specific detectivity

The irradiance impacts perpendicular on the photodetector through free space. Here $R = \frac{{I_{\mathrm{ph}}}}{{E_{\mathrm{e}}A}}$ ≈ 0.6 A W^–1^, where *I*_ph_ is the photocurrent (3.3 pA at a drain bias of 50 mV), *E*_e_ is the irradiance (130 μW cm^−2^ at a wavelength of 1,800 nm) and *A* is the area where the electrodes overlap (60 nm × 70 μm). The specific detectivity is $D^\ast = \frac{{\frac{R}{{I_{\mathrm{ds}}}}\sqrt A }}{{\frac{{i_\mathrm{n}}}{{I_{\mathrm{ds}}}}}}$ ≈ 3 × 10^9^ Jones, where *I*_ds_ is the drain current (9.2 × 10^−11^ A) and *i*_n_/*I*_ds_ is the current-normalized noise current (4 × 10^−4^ Hz^−0.5^).

### Toxic and environmental concerns of mercury-containing devices

If the complete waveguide surface area (<100 μm × 100 μm) is covered with a 180-nm-thick HgTe film (not taking QD ligands and packing density of the QDs into account), it would result in a volume of 1.8 × 10^−9^ cm^3^ HgTe equivalent to about 10 ng of mercury. This is far less compared with canned tuna (about 200 μg kg^–1^ on average)^33^. Although our device requires a reasonably small amount of highly toxic mercury, we hope that in the long run, all the toxic elements in the device will be replaced with more benign elements. Furthermore, the release of mercury into the environment should be minimized by implementing rigorous recycling protocols.

## Online content

Any methods, additional references, Nature Research reporting summaries, source data, extended data, supplementary information, acknowledgements, peer review information; details of author contributions and competing interests; and statements of data and code availability are available at 10.1038/s41566-022-01088-7.

## Data Availability

Source data are provided with this paper. All other data are available from the corresponding authors upon reasonable request.

## Source data

Source Data Fig. 2 Spectral response of the photodetector, QD absorption in solution and noise measurement.
Source Data Fig. 3 Unprocessed measurement data and waveguide spectrometer with one light source.
Source Data Fig. 4 Unprocessed measurement data and waveguide spectrometer with two light sources.
Source Data Extended Data Fig. 7 Calibration of piezo-stage by a waveguide spectrometer at different wavelengths.
Source Data Extended Data Fig. 8 Comparison of light modulation by an optical chopper and mirror vibration modulation.
Source Data Extended Data Fig. 9 Plot of raw data; same source data as Extended Data Fig. 8.
